# Antenatal Ultrasound Findings in Spinal Muscular Atrophy Type 0

**DOI:** 10.1002/mgg3.70128

**Published:** 2025-09-09

**Authors:** Stephanie Stokes, Madeline Snipes, Lee D. Moore, Natalia Schlabritz‐Lutsevich, Vidalin Amy, James Maher

**Affiliations:** ^1^ Department of Obstetrics and Gynecology Augusta University Augusta Georgia USA; ^2^ Augusta University School of Medicine Augusta Georgia USA; ^3^ Texas Tech Health Science Center Texas USA; ^4^ Permian Basin Advanced Fertility Centers USA; ^5^ Department of Maternal‐Fetal Medicine Augusta University Augusta Georgia USA

**Keywords:** antenatal ultrasound, fetal cardiac anomalies, genetic screening, prenatal diagnosis, severe spinal muscular atrophy, spinal muscular atrophy type 0

## Abstract

**Introduction:**

Spinal muscular atrophy (SMA), caused by pathogenic variants in the survival motor neuron (*SMN*) gene, is the most common genetic cause of mortality in children under the age of two. Prior reports of obstetric sonograms performed in pregnancies with severe forms of fetal SMA have discrepant findings that may stem from a failure to account for the *SMN2* copy number.

**Methods:**

We present a neonate diagnosed with SMA type 0 postnatally (*0SMN1/1SMN2* genotype). Antenatally, the fetus was noted to have HLHS (hypoplastic left heart syndrome), 2:1 AV block (atrioventricular), thickened nuchal translucency, polyhydramnios, and perceived maternal decreased fetal movement, and the mother declined genetic testing.

A literature search was conducted to analyze potential prenatal findings in severe SMA type 0.

**Results:**

The most common associations from 32 cases of SMA type 0 include cardiac defects, increased NT (nuchal translucency), decreased fetal movement, and contractures noted postnatally. Other associations that were present in the literature and in our case include nonvertex presentation, polyhydramnios, and fractures after birth.

**Conclusion:**

Prenatal onset SMA type 0 with one copy of *SMN2* appears to have a distinct phenotype. Cardiac anomalies, increased nuchal translucency, and decreased maternal perception of fetal movement in the third trimester are the most frequent findings, and if found, should prompt SMA testing.

## Graphical abstract

Antenatal ultrasound shows a distinct pattern of findings in fetuses affected by Spinal Muscular Atrophy type 0. The findings of cardiac anomalies, increased nuchal translucency in the first trimester, and an increased nuchal skin fold thickness in the second trimester should prompt screening for SMA. Third trimester polyhydramnios and decreased fetal movement as well as post‐natal long bone fractures are also commonly seen.

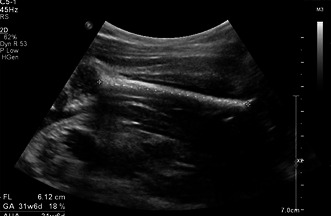

## 1 Introduction

Spinal muscular atrophy (SMA) [OMIM # 253300] is an autosomal recessive neuromuscular disorder characterized by progressive spinal motor neuron degeneration. It is one of the most common genetic causes of child death under the age of 2 (Carré and Empey 2016). SMA occurs in one in 6000 to 10,000 live births. Before the availability of molecular genetic testing, most cases were identified by postnatal clinical evaluation. The finding of a single *SMN1* copy number on dosage analysis detects over 90% of SMA carriers in all ethnic groups except African Americans due to increased 2 + 0 cis configuration in *SMN1* (Carré and Empey 2016; Mercuri et al. 2018; Rouzier et al. 2020).

SMA is an autosomal recessive disorder caused by survival motor neuron (*SMN*) gene mutations on chromosome 5 due to homozygous loss of *SMN1*, with the severity of phenotypic presentation dependent upon *SMN2* copy number. Humans have at least two highly homologous copies of the *SMN* gene on each chromosome (*SMN1* and its centromeric homolog *SMN2*), which are mapped to chromosome locus 5q13 in a large (500 kb) inverted duplication region (Chaudhary et al. 2022; Motyl et al. 2020). The *SMN2* gene is a pseudogene that differs from *SMN1* by five nucleotides. The *SMN2* gene produces about 10% of the functional *SMN* protein due to a mutation in the splice region in exon 7, which affects the assembly of the final *SMN* messenger RNA. There is an inverse relationship between disease severity in SMA and the *SMN2* copy number in affected individuals, such as severely affected SMA type 0 patients (Butchbach 2016). The telomeric *SMN1* gene is the SMA‐determining gene, and the centromeric *SMN2* gene copy number modulates the observed phenotype of SMA in affected individuals. The *SMN* gene product is a 38 kDa survivor motor neuron protein found throughout the cytoplasm and nucleus. It involves critical cellular functions, including RNA processing and cytoskeleton dynamics (Chaytow et al. 2018).

The *SMN* gene product is integral to many embryological developmental stages, including cardiac, vascular, and bone development, which have been demonstrated across multiple SMA murine models (Eshraghi et al. 2016; Hsieh‐Li et al. 2000; Osman et al. 2019; Shanmugarajan et al. 2009; Sleigh et al. 2011; Wan et al. 2018).

Antenatal findings in ultrasound literature have shown disagreements about the presence and types of sonographic findings in SMA‐affected fetuses. These discrepancies likely stem from a failure to account for copy numbers of the *SMN2* genes in affected neonates.

In human**s**, osteopenia, contractures, and fractures have been reported in infants diagnosed with severe SMA type 0 and only a single *SMN2* copy number (Baranello et al. 2019; Courtens et al. 2002; Čupāne et al. 2023; García‐Cabezas et al. 2004; Grotto et al. 2016; Jain et al. 2019; Markowitz et al. 2004). In contrast, fetuses with SMA type 1 with at least two copies of *SMN2* (classic Werdnig–Hoffman) generally do not have phenotypic findings at birth and may escape antenatal detection. Such infants usually become symptomatic during the first postnatal months with proximal muscle weakness, a weak cry, and progressive breathing difficulties. Therefore, it is unlikely that a prenatal ultrasound will reveal SMA‐related abnormalities. SMA type 0‐affected fetuses carry only one copy of the *SMN2* gene, and their anatomic ultrasounds may demonstrate features that herald the prenatal onset of disease (Balslev et al. 2001; Čupāne et al. 2023; Dakhoul 2017; Dubowitz 1999; Dubowitz 2019; Erbas and Gusset 2021; García‐Cabezas et al. 2004; Grotto et al. 2016; Kitaoka et al. 2020; Parra et al. 2010; Parra et al. 2011; Singh et al. 2021; Tiberi et al. 2020; Zadeh et al. 2011). SMA with no copies of *SMN2* is lethal during embryonic development (Farrar and Kiernan 2015). Review of the case reports and case series of SMA type 0 patients documents a previously unappreciated and distinctive clinical phenotype characterized by an increased incidence of several antenatal findings, including cardiac structural abnormalities, increased nuchal translucency, polyhydramnios, and malpresentation. We present a case of SMA type 0 and review the literature of antenatal findings of SMA.

## 2 Materials and Methods

### 2.1 Ethical Compliance

This study was determined to be exempt from further review by the ethics committee as there is deidentified patient information and the patient has granted consent to present the case below, available upon request.

### 2.2 Authorship

All listed authors qualify for authorship. All authors made substantial contributions to the conception and design of the manuscript and have been involved in drafting the manuscript or revising it critically for important intellectual content. All authors have given final approval for the version to be published. Each author participated in the work, takes public responsibility for the content, and agreed to be accountable for all aspects of the work in ensuring that questions related to the accuracy or integrity of any part of the work are appropriately investigated and resolved.

We present a case of a neonate with respiratory distress and hypotonia diagnosed with SMA type 0 after molecular testing showed homozygous deletion of *SMN1* and one copy of *SMN2*. HLHS (hypoplastic left heart syndrome), 2:1 AV block (atrioventricular), thickened nuchal translucency, polyhydramnios, and perceived decreased fetal movement were seen. Prenatal genetic testing was offered and declined.

A literature review used English keywords: “Spinal muscular atrophy, antenatal ultrasound findings, cardiac anomalies, severe spinal muscular atrophy, survival motor neuron gene, decreased fetal movement.” Ten case reports, 4 retrospective studies, 3 case series, and 2 prospective studies met our criteria and were accessible to be reviewed to analyze potential prenatal findings in severe SMA with 1 copy of *SMN2*. GenBank reference sequence and version numbers were verified for the human genes described in our case and literature review: NM_000344.4 for SMN1 and NM_017411.4 for *SMN2*.

*In total, our literature review identified 32 cases of molecularly confirmed SMA type 0 (0SMN1/1SMN2) shown in* Table 1. Not *all studies specifically commented on* the *evaluation of the phenotypic findings in question, their absence/presence, and the diagnosis timing made if present (antenatal, postnatal). Therefore, we will briefly describe the total number of cases in each category*, the *percentage of those cases that comment on the finding(absent/present), for CHD, increased NT, polyhydramnios, contractures, fractures, fetal malpresentation, and maternal perception of decreased fetal movement. We will then discuss the timing (antepartum* vs. *postpartum) The following sections summarize the findings in these 32 cases of molecularly confirmed SMA type 0 and assist with* the *interpretation of* Tables 1 and 2.

**TABLE 1:** Summary of prenatal/postnatal findings of SMA from prior literature review.

Phenotypic finding	Total cases	Prenatal diagnosis	Postnatal diagnosis	TA	Percentage
Congenital heart disease	23	7	12	19	83%
Thickened NT	16	10	N/A	10	63%
Polyhydramnios	25	6	NA	6	24%
Fetal malpresentation	19	7	NA	7	37%
Fractures	18	0+	5	5	28%
Contractures	32	1++	25	25	78%

**TABLE 2:** Prenatal and postnatal findings of SMA from prior literature review.

Ref	Study	Sex	NT	Poly	Heart defect (pre/post)	DFM	Nonvertex	Fracture (pre/post)	Contracture (pre/post)	SMN2 Copy #
Čupāne et al. (2023)	Case report	F	+	+	−/+	+	+	NS/+	NS/−	1
Matesanz et al. (2020)	Case report	F	+	−	+/+	−	−	NS/+	NS/−	1
Kitaoka et al. (2020)	Case report	M	NS	NS	NS/−	+	+	−/−	NS/+	2
Jain et al. (2019)	Case report	M	NS	+	NS/NS	+	+	−/+	−/+	1
Singh et al. (2018)	Case report	F	−	+	NS/NS	+		−/−	−/+	NS
Dakhoul (2017)	Case report	M	NS	+	NS/NS	+	NS	NS/NS	NS/NS	NS
Grotto et al. (2016)	Retrospective review (16 patients with SMA 0)	10F/6M	** + 7/13 (Only 13 had NT measured)	0/16	**(+ 2/13)/(+ 9/13)** (Only 13 had pre and postnatal cardiac evaluation)	+9/16	+5/16	NS/(1/14)** (Only 14 patients had fractures assessed)	**(+1/13)/(+15/16) (Only 13 patients had this assessed prenatally)	1
Barone and Bianca (2013)	Retrospective review (29 patients with untyped SMA)	NS	0/29	NS	(−0/29)/(−0/29)	NS	NS	NS	NS	NS
Parra et al. (2012)	Prospective study of 98 patients (19 with SMA)	NS	* + 1/19	NS	*(+ 1/19)/(1+/19)*	NS	NS	NS	NS	*(1/19) ‐ 1 (18/19)‐ 2
Zadeh et al. (2011)	Retrospective study (12 with untyped SMA)	NS	0/12	NS	NS	NS	NS	NS	NS	NS
Parra et al. (2011)	Prospective study 29 patients (7 with SMA between 11 and 14 wga)	4F/3M	NS	NS	NS	0/7	NS	NS	NS	(1/7)‐ 1 (6/7)‐ 2
Rudnik‐Schöneborn et al. (2008)	Retrospective study (65 patients with SMN met criteria)	NS	NS	* + 3/4	Not specific pre/post *(+3/65)	* + 2/4	NS	NS	NS	*(4/65)‐1 (59/65)‐ 2 (3/65)‐ 3
Menke et al. (2008)	Case report (2 siblings with presumed SMA1)	1F/1M	NS(+1/1)	*NS	NS(+1/1)/(2+/2)**	+2/2	*NS	−/−	NS/(2+)	NS
García‐Cabezas et al. (2004)	Case report	M	NS	+	NS/+	+	NS	NS/+	NS/+	1
Macleod et al. (1999)	Case series (5 cases)	5 M	NS	NS	(+0/5)/(+2/2)**(Only 2 had comments on postnatal cardiac exams)	+5/5	NS	NS	NS/(2/5)	1
Kelly et al. (1999)	Case report	M	NS	NS	NS	NS	+	NS/+	NS	NS
Jong et al. (1998)	Case report	M	NS	NS	+	NS	NS	NS	−/−	NS
Devriendt et al. (1996)	Case report	M	NS	−	NS/+	+	NS	NS	NS	1
Mulleners et al. (1996)	Case report (2 patients with presumed SMA1 and SMA3)	*NS/1 M	NS	NS	NS/(+2/2)	NS	NS	NS	NS/(+1/1)**	NS

*Note:* Summary of the antenatal and postnatal findings seen in prior case reports, case series, retrospective reviews, and prospective studies about SMA. All cases had molecularly confirmed SMA.

Abbreviations: **, examination not done on all patients; *, patient/patients with 1 copy SMN2; –, not present; +, present; DFM, decreased perceived fetal movement; F, female; homo, homozygous; M, male; NF, nuchal fold thickness; NS, not stated; NS, not stated; NT, nuchal translucency; poly, polyhydramnios; post, after delivery; pre, prior to delivery; Ref, reference; SMNc, centromeric copy of spinal muscular atrophy (SMN2).

Prenatal ultrasound and specific details on the presence or absence of cardiac defects were reported in 23/32 cases, with 4/23 (17%) cases detected antenatally, and an additional 3 cases documented by Rudnik‐Schöneborn et al. (2008) did not specify if the diagnosis was made antenatally or postnatally. If those cases were included, cardiac defects would have been diagnosed in 7/23 cases (30%). Postnatally, a total of 16/23 (70%) or 19/23 (83%) cases, including the 3 Rudnick et al. cases, showed evidence of cardiac defects (Table 1). Defects ranged from mild atrial septal defects (ASDs) to more significant ASDs, ventricular septal defects (VSDs), and severe cardiac anomalies such as HLHS.

The presence or absence of polyhydramnios was reported in 25/32 cases, with polyhydramnios being present in 6/25 (24%) cases. The NT was reported in 16/32 cases. Of the 16 cases documented, 10/16 (63%) reported increased NT. The presence or absence of fractures was documented in 18/32 cases, with only Jain et al. (2019) explicitly saying that there was no evidence of fractures seen antenatally. Postnatally, 5/18 (28%) identified fractures.

The presence of contractures postnatally was reported in 25/32 cases, with 20/25 (80%) being seen in the immediate post‐natal period. In 14/32 cases, antenatal evaluation was reported, with only 1/14 (7%) case of contractures being diagnosed antenatally. Fetal presentation was reported in 19/32 cases, with 7/19 (37%) showing fetal malpresentation. Maternal perception of fetal movement was reported in 30/32 cases, with 20/30 (67%) patients reporting decreased fetal movement in the third trimester. Parra et al. (2011) had one patient with 1 copy of *SMN2*, but no maternal perception of decreased fetal movement was assessed since this was a first trimester report (Parra et al. 2011).

## 3 Case Description

**IMAGE 1:**
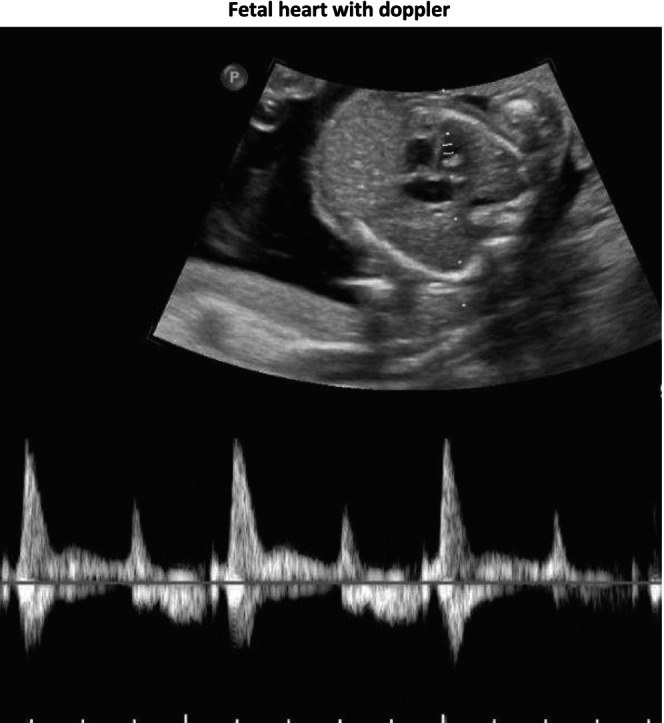
Lateral 4 chamber fetal view showing second‐degree atrioventricular (AV) block and hypoplastic left heart syndrome (HLHS).

**IMAGE 2:**
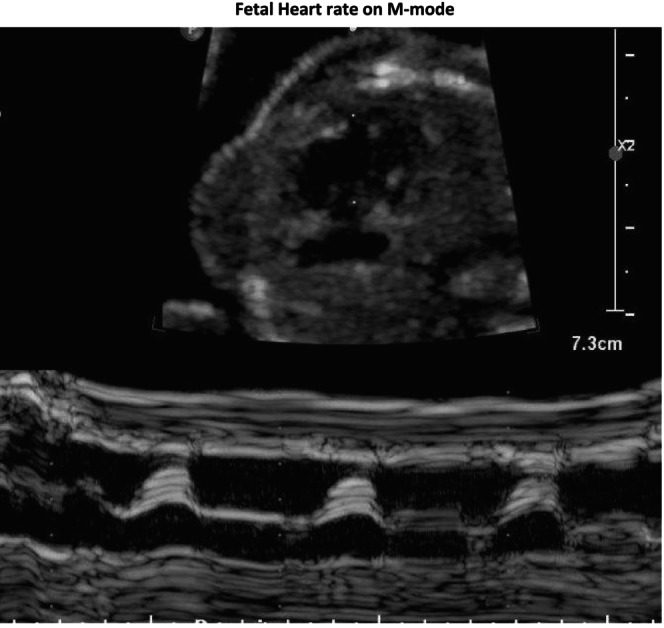
Fetal heart rate on M‐mode.

**IMAGE 3:**
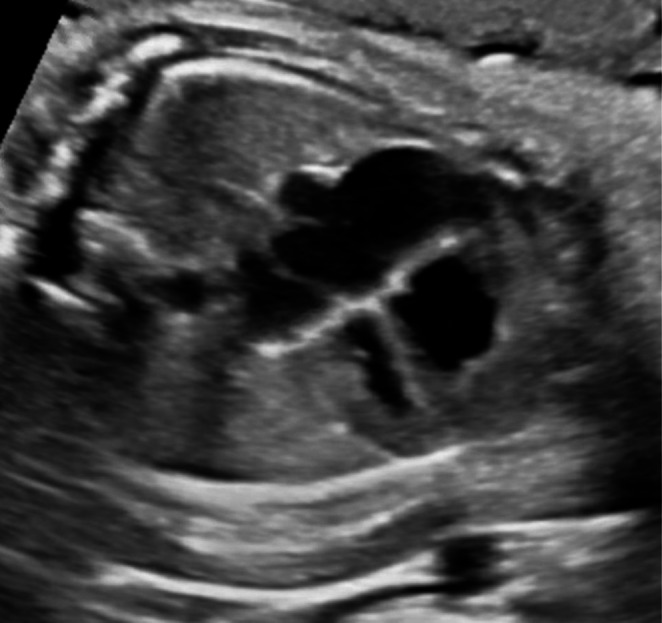
Four‐chamber heart hypoplastic left heart.

**IMAGE 4:**
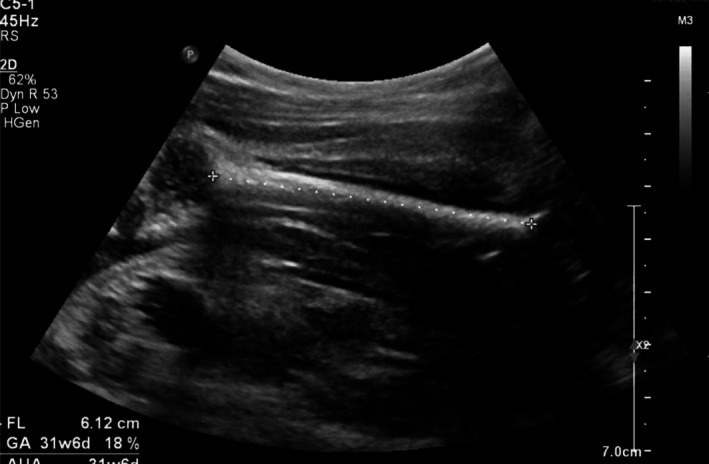
Fetal femur at 31 weeks fetal femur, no sign of fracture.

A 24‐year‐old G4P2012 at 16.2 weeks of gestation (WGA) with no known familial history of SMA or prior affected children with SMA presented for MFM consultation after numerous cardiac abnormalities and fetal arrhythmia were seen on ultrasound. The patient had NIPT performed at 12 WGA, revealing a male fetus with a low risk for Sex Chromosome Aneuploidy, T18, T21, and T13. She declined gene carrier testing, and after a detailed anatomic ultrasound showed hypoplastic left heart syndrome and intermittent 2:1 AV block (Images 1, 2, 3, 4), the patient declined amniocentesis. Antenatal testing started at 31 WGA with a BPP due to a persistent 2:1 AV block. At 37 WGA, the patient had a BPP of 6/10 (points removed for movement and tone), and bi‐weekly BPPs were started with new findings of polyhydramnios and breech presentation noted. The patient presented at 38.3 WGA, and a prolonged bradycardic episode was seen on the fetal monitoring. An emergency cesarean delivery was performed, and the newborn, weighing 2340 g, had APGAR scores of 4, 5, and 7 at one, five, and ten minutes, respectively. He required intubation due to poor respiratory effort. After transferring to the neonatal intensive care unit, X‐rays showed fractures of the left and right humeri and left femur. There were no rib fractures or findings suggestive of skeletal dysplasia. An echocardiogram demonstrated a hypoplastic left heart, an atrial septal defect, and Mobitz type 1 heart block. Tongue fasciculation and hypotonia were present at birth. Electromyogram and nerve conduction studies showed denervation in multiple peripheral and cranial nerves concerning SMA. Genetic testing demonstrated SMA type 0 with homozygous deletion of the *SMN1* gene and one copy of *SMN2*. The infant was not a candidate for gene therapy due to high titers of adenovirus associated with AAV9 antibodies (>/=1:200). On DOL 20, a 3:2 heart block was diagnosed. After family consultation with palliative care, the care plan was redirected to comfort care status. The infant continued to deteriorate and died at 27 days of age.

## 4 Discussion

Reports of antenatal ultrasound findings in SMA are inconsistent across the spectrum of type 0 to type III. However, we believe that the literature suggests that the lower the *SMN2* copy number, the more likely it is to detect ultrasound abnormalities. When limiting our review to only SMA cases with molecular testing demonstrating a single copy of the *SMN2* gene, as shown in Tables 1 and 2, there appears to be an increased incidence of phenotypic findings in fetuses with SMA Type 0, which has not been previously summarized. The prevalence of cardiac anomalies in fetuses with SMA 0 is higher than in the general population. Increased nuchal translucency, polyhydramnios, malpresentation, and decreased maternal perception of fetal movement, as shown in Tables 1 and 2 are also frequently noted. Multiple neonatal fractures after an atraumatic delivery and/or joint contractures further raise concern for an affected fetus and should prompt consideration for the diagnosis of SMA Type 0.

*The following sections will describe the 32 cases of molecularly confirmed SMA type 0* (*0SMN1/1SMN2*) *shown in* Tables 1 and 2.

### 4.1 Cardiac Anomalies and Increased Nuchal Translucency

*Multiple investigators have described the relationship between SMA and the increased prevalence of cardiac anomalies and increased NT in retrospective and prospective studies, case series, and case reports which are listed in* Tables 1 and 2. In 1999, Macleod et al. presented a case series of 5 patients with presumed type 0 SMA. All patients reported decreased fetal movement in the third trimester. Postnatally, all infants manifested with severe respiratory insufficiency and hypotonia. Cardiac defects including ASD and ASD with mitral valve hypoplasia were seen in 2/5 patients. The authors concluded that this was a severe form of SMA, which had an even lower *SMN2* gene dosage than previously seen in SMA type 1.

A retrospective study of 65 patients reported by Rudnik‐Schöneborn et al. (2008) evaluated the incidence of congenital heart defects in patients with SMA. The researchers found 4/65 patients had one *SMN2* copy number, and 3/4 of these patients had hemodynamically significant cardiac defects. The study did not comment on diagnosis timing and whether diagnosis was antenatal vs. postnatal. The authors believe that SMA with a single *SMN2* copy number showed cardiac defects at a greater than expected frequency.

Parra et al. (2012) performed a prospective study of 98 patients at risk for SMA. After molecular testing by CVS, 19 were diagnosed with SMA. Eighteen fetuses had two copies of the *SMN2* gene, and all had normal NT and cardiac exams. One affected fetus had only a single copy of the *SMN2* gene and had abnormal antenatal findings of increased NT and HLHS.

Zadeh et al. (2011) 7509‐7514 reported a retrospective study of 12 patients with untyped SMA who had first trimester NT measurements performed. All 12 patients had normal NT measurements. The authors concluded that increased NT is not associated with SMA. These cases were likely mild forms of SMA as most diagnoses were made after DOL 30. The *SMN2* copy number was not reported.

Barone and Bianca (2013) performed a retrospective observational study of 29 women who had a fetus or child with SMA and underwent first trimester NT assessment. The study found no increased NT, antenatal malformations, and concluded that there was no association between SMA and ultrasound findings. All patients with SMA had homozygous deletion of *SMN1*. However, they did not document *SMN2* copy number.

Grotto et al. (2016) performed a retrospective review of 16 patients with SMA; 13/16 had NT measurements and postnatal echocardiograms. Cardiac defects were present in 9/13 patients. Two patients had small to moderate ASDs, while the other seven had either a larger VSD, large ASD, and/or total anomalous pulmonary venous return. Increased nuchal translucency was found in 7/13 patients.

As shown in Table 2, two additional case studies showed evidence of increased NT and cardiac defects. VSD was diagnosed prenatally in one patient, while ASD was diagnosed postnatally in the other (Čupāne et al. 2023; Matesanz et al. 2020). *In total*, *NT was assessed in 16/32 cases*, *with 10/16 showing abnormal NT measurement. CHD was mentioned in 23/32 cases*, *with 19/32 being found to have evidence of CHD* (Table 1).

### 4.2 Decreased Perceived Fetal Movement, Malpresentation, and Polyhydramnios

Many SMA‐affected neonates with severe SMA have been reported in the literature to be associated with decreased fetal movement, polyhydramnios, and malpresentation, as shown in Table 1.

Macleod et al. (1999) report 5 patients who reported decreased fetal movement and were diagnosed postnatally. The diagnosis of SMA with reduced *SMN2* copy number was made by *SMN:MPZ* ratio in this study. The authors concluded that this was a more severe form of SMA than previously seen (presumptive Type 0).

Rudnik‐Schöneborn et al. (2008) performed a retrospective study of 65 patients with SMA. The researchers found 4/65 cases had 1 copy of *SMN2*. Decreased fetal movement was reported in 2/4 patients with SMA, and 3/4 had polyhydramnios (Singh et al. 2021).

A prospective study by Parra et al. (2012) was then conducted on 29 pregnancies at risk for severe SMA to assess first trimester fetal movement in SMA. SMA was diagnosed in 7/29 patients, with 1/7 patients having 1 copy of *SMN2*. Using 2D ultrasound between 11 and 14wks to qualitatively evaluate the occurrence of generalized body movements, isolated movements of arms and legs, head movements, startles, and hiccups in SMA‐affected (including SMA type 0) and unaffected neonates (Parra et al. 2011). The authors concluded that using 2D ultrasound, data do not detect limitations of movements in fetuses with SMA in the first trimester. Most patients discussed in Table 1 report that decreased fetal movement is seen in the third trimester between 32 and 36 wga.

A retrospective study by Grotto et al. of 16 patients with SMA type 0 reported decreased fetal movements in 5/16 between 32 and 36 wga (Grotto et al. 2016). Aside from the retrospective and prospective studies, multiple case reports have shown SMA type 0 affected pregnancies with decreased fetal movement, polyhydramnios, and breech presentation (Čupāne et al. 2023; Dakhoul 2017; García‐Cabezas et al. 2004; Jain et al. 2019; Kitaoka et al. 2020; Matesanz et al. 2020; Menke et al. 2008; Singh et al. 2018).

In total 19/32 cases documented fetal presentation, with 7/19 being found to have fetal malpresentation at the time of delivery. Amniotic fluid index or maximum vertical pocket were reported in 25/32 cases, with 6/25 being diagnosed with polyhydramnios. Fetal movement was discussed in 30/32 cases, with 20/30 reporting decreased fetal movement.

### 4.3 Neonatal Fractures, Contractures, and Osteopenia

*Bone pathology has been observed in multiple studies and case series*. A case series by Macleod et al. (1999) reports that 2/5 patients with SMA type 0 had congenital contractures after birth. No fractures were noted. A case report by García‐Cabezas et al. (2004) describes a neonate born via cesarean section found to have multiple fractures, joint contractures, and respiratory insufficiency following delivery. Postnatally, molecular testing in this case revealed *SMN* with a single copy of *SMN2*.

Grotto et al. (2016) commented on contractures and fractures in 16 cases of SMA type 0. All 16 patients had some degree of joint contracture. Joint contractures were noted in 1 patient prenatally, with 15/16 not being appreciated until immediately after delivery. Fractures were evaluated in 14/16 patients, and 1/14 patients with SMA type 0 had evidence of a postnatal fracture after delivery. Jain et al. (2019) describe a case report of a woman with decreased fetal movement who was found after birth to have a neonate with contractures and femur fractures. Postnatal SMA type 0 was diagnosed.

Most recently, 2 case reports have documented SMA type 0 infants being diagnosed postnatally with fractures; however, congenital contractures were not reported in these cases (Čupāne et al. 2023; Matesanz et al. 2020). In total, 18/32 cases discussed the presence/absence of fractures, with 5/18 being found to have evidence of fractures (all diagnosed postnatally). Contractures were seen in all 26/32 cases (1 case noted prenatally). Even when fractures were not noted at birth, SMA type 0 infants have been found to have generalized osteopenia (Singh et al. 2021). The increased findings of fetal fractures could be the result of decreased fetal movement contributing to osteopenia. Contractures further lead to increased fracture risk. *Animal studies have shown the SMN1 protein to be integral to bone formation* (Shanmugarajan et al. 2009).

### 4.4 SMA Classification

Before the advent of molecular testing, SMA was grouped into discrete subtypes. It is now clear that the phenotype of SMA associated with *SMN1* pathogenic variants includes a broad spectrum of disease. Newly approved treatment options are further changing the natural history of SMA phenotypes and further blurring the boundaries between subtypes (Kong et al. 2021). Nonetheless, the existing classification system based on the age at symptom onset and maximum function attained with supportive care is helpful for prognosis and management (Singh et al. 2018). The current classification scheme includes five subtypes of SMA resulting from the homozygous deletion of the *SMN1* gene and between 1 and 8 copies of the *SMN2* gene, with lower *SMN2* copy number causing more severe forms of SMA (Kong et al. 2021).

## 5 Treatment

Gene replacement and pre‐mRNA splicing modifier therapies represent breakthrough gene‐targeting treatments for spinal muscular atrophy (SMA). Animal model evidence shows that in utero treatment of SMA results in enhanced *SMN* expression during embryonic development and subsequent post‐natal clinical symptom improvement. Treatment with a small‐molecule *SMN2* splice modifier increases the production of the *SMN* gene product from the *SMN2* gene, which results in better motor unit development, motor axon function, and short‐ and long‐term motor improvements in SMA mice (Day et al. 2022). Since 2016, therapeutic interventions for SMA have been clinically available in humans. Increases in *SMN* gene protein production in human trials are associated with improved outcomes if the treatment is started before symptom onset (Motyl et al. 2020).

These treatments may prevent the development or slow the progression of some features of SMA. Treatment for SMA type 0 is still debated, but there is currently little evidence of benefit to postnatal treatment (Barone and Bianca (2013; Dakhoul 2017; Eshraghi et al. 2016; Motyl et al. 2020; Shanmugarajan et al. 2009; Singh et al. 2021).

ACOG guidance recommends offering prenatal screening for common genetic conditions and aneuploidy (Dyr et al. 2019). Our patient was offered amniocentesis after consultation for the abnormal antenatal findings but declined both genetic screening and invasive testing. Antenatal diagnosis would have allowed informed decisions about the prognosis for pregnancy and increased psychological support for the family. It is not yet known if in utero treatment for SMA type 0 will alter the generally poor prognosis for this condition (Day et al. 2022; Erbas and Gusset 2021).

## 6 Conclusion

*The association between increased nuchal translucency and fetal cardiac disease is well known; SMA type 0 should also be added as a differential diagnosis for both findings. Clinical suspicion for this disease may further be enhanced by subsequent findings of maternal perception of decreased fetal movement, polyhydramnios, and malpresentation in the third trimester*, *prompting further discussion about testing for this condition if not previously performed*.

## Author Contributions

The listed authors qualify for authorship. All authors contributed substantially to the conception and design of the manuscript and have been involved in drafting it or revising it critically for important intellectual content. All authors have given final approval for the version to be published. Each author participated in the work, takes public responsibility for the content, and agreed to be accountable for all aspects of the work in ensuring that questions related to the accuracy or integrity of any part of the work are appropriately investigated and resolved.

## Consent

Patient consent has been given to publish the case report, which is available upon request.

## Conflicts of Interest

The authors declare no conflicts of interest.

## Data Availability

Data sharing not applicable ‐ no new data generated, or the article describes entirely theoretical research.
